# Towards Interband Cascade lasers on InP Substrate

**DOI:** 10.3390/ma15010060

**Published:** 2021-12-22

**Authors:** Krzysztof Ryczko, Janusz Andrzejewski, Grzegorz Sęk

**Affiliations:** Department of Experimental Physics, Faculty of Fundamental Problems of Technology, Wrocław University of Science and Technology, Wybrzeże Wyspiańskiego 27, 50-370 Wroclaw, Poland; janusz.andrzejewski@pwr.edu.pl (J.A.); grzegorz.sek@pwr.edu.pl (G.S.)

**Keywords:** interband cascade laser, type II quantum well, indium phosphide, mid infrared, optical gain

## Abstract

In this study, we propose designs of an interband cascade laser (ICL) active region able to emit in the application-relevant mid infrared (MIR) spectral range and to be grown on an InP substrate. This is a long-sought solution as it promises a combination of ICL advantages with mature and cost-effective epitaxial technology of fabricating materials and devices with high structural and optical quality, when compared to standard approaches of growing ICLs on GaSb or InAs substrates. Therefore, we theoretically investigate a family of type II, “W”-shaped quantum wells made of InGaAs/InAs/GaAsSb with different barriers, for a range of compositions assuring the strain levels acceptable from the growth point of view. The calculated band structure within the 8-band k·p approximation showed that the inclusion of a thin InAs layer into such a type II system brings a useful additional tuning knob to tailor the electronic confined states, optical transitions’ energy and their intensity. Eventually, it allows achieving the emission wavelengths from below 3 to at least 4.6 μm, while still keeping reasonably high gain when compared to the state-of-the-art ICLs. We demonstrate a good tunability of both the emission wavelength and the optical transitions’ oscillator strength, which are competitive with other approaches in the MIR. This is an original solution which has not been demonstrated so far experimentally. Such InP-based interband cascade lasers are of crucial application importance, particularly for the optical gas sensing.

## 1. Introduction

An important class of coherent radiation sources in the mid-wave infrared have become the interband cascade lasers (ICLs) employing type II, indirect in the real space optical transitions [1,2]. The concept of ICL is beneficial with respect to some of the device performances, i.e., it combines a voltage-efficient cascading scheme, borrowed from the quantum cascade lasers, and a long upper-level recombination lifetime as in a conventional diode laser. The ICLs are usually based on a broken gap material system of InAs/GaInSb [1,2,3,4], which fulfills the electronic structure and technological requirements, and simultaneously allows to reach a broad range of mid infrared (MIR) [5]. The ICLs have already been demonstrated to operate in continuous wave and single mode at room temperature, or even above, in the spectral range from below 3 to above 6 μm [6,7,8,9,10,11] and record low power consumption (<0.1 W at threshold at 300 K), ultralow threshold current densities as for MIR lasers [6,12] as well as high cw output powers [4]. These characteristics translate into numerous applications of ICLs, including in optical gas sensing [13,14], multi-gas analyzers [15], industrial process control [16,17], environmental pollution monitoring [18], medical diagnostics [19], infrared countermeasures [20], gas leakage detection [21], free space optical communication [22] with demonstrations of the efficient optical wireless link for military applications [23], combustion diagnostics [24], IR scene projection [25], and detection of explosives [26].

Current ICLs are grown by molecular beam epitaxy on GaSb or InAs substrates. In spite of all their abovementioned achievements, there remain applications-driven further demands to improve the active material and the device characteristics or to reduce the fabrication costs. These would potentially be obtainable when ICLs could be grown in mature and cheaper technologies based on GaAs or InP. As for the case of GaAs-substrate some preliminary demonstrations exist—an ICL in a pulsed mode and low temperatures (up to 270 K) has been reported [27], such are still missing for the case of InP-based technology. The latter, which is also cost-effective, offers additional advantages like simplified epitaxial structure, because the InP can also serve as an optical cladding layer, improved heat dissipation material or allows considering the option of using a high-power near-IR sources to optically pump an epitaxial-side-down-mounted device through a transparent substrate [28]. Overall, such a novel ICL would be a breakthrough solution because InP-substrate-based laser devices of various architectures have only been demonstrated significantly below 3 μm [29,30,31,32], with slightly longer wavelengths achieved in photoluminescence measurements (PL) [29,32,33,34] (see also Table 1 where the current status of the InP-based quantum well (QW) structures predicted for emission above 2 μm is summarized), including an example of spontaneous emission from a type II GaInAs/GaAsSb QW at 3.9 μm [32].

The latter example indicates the potential of this material’s’ combination among other proposed solutions for the MIR range. There have been reported a couple of simulated designs of type I or type II lasers’ active regions involving various materials matching the InP technology to extend the emission wavelengths [28,33,35,36,37], but they have never been translated into operational devices. The reasons they have not been successful are manifold: the proposed materials are of insufficient structural or optical quality (e.g., the dilute nitrides or dilute bismides), the electronic confinement of one type of carriers is too weak, or the strain is too high, or a combination of several of these. Therefore, searching for new solutions is still necessary—within the considered systems, the one employing InGaAs/GaAsSb seems to stand out.

In our studies, we have mainly been inspired by the work from [33], which had shown that InP-based type-II InGaAs/GaAsSb heterostructures are promising for emission or detection for λ around 2 μm, plus that they offer relatively low net strain, i.e., more favorable material growth that allows to avoid the potential strain relaxation. The main objective of our current work is to propose such a type II active area QW structure that would allow emission in the application-relevant range, which could further be directly used to fabricate operational ICLs of that kind in low-cost, mature InP-substrate-based technology. We propose several designs of the active region made of InGaAs, GaAsSb and AlAsSb layers and model the respective electronic and optical properties. We mainly concentrate on extension of the emission wavelength to the range of about 3–5 µm, because it is especially relevant for applications in the optical laser-based gas sensing, as many of the environmentally or medically important gasses have their strongest absorption lines there. It is also one of the windows of the free space optical communication. We originally propose to include a thin InAs layer, which becomes an additional tuning knob to tailor the electronic structure and to reach easier the longer wavelengths of above 4 μm. We demonstrate for the first time that using the approach with such an especially modified type II InGaAs/GaAsSb active region compatible with an InP substrate should allow the fabricating of the interband cascade lasers emitting in the target MIR region, with expected performances competitive to the state of the art devices on GaSb substrate, meanwhile being significantly cheaper in production.

## 2. Materials and Methods 

We began the considerations with calculating the band structure and wave functions near the center of the Brillouin Zone. The eight-band k·p model within the envelope function approximation was used to calculate the electron and hole energy levels (subbands) in an active region of an ICL on InP substrate. The total Hamiltonian for the strained QW, which describes the energy spectrum for both conduction and valence subbands, is given by [38]

$$H=H_{8\times 8}+H_{S}+V\left(z\right),$$

where $H_{8\times 8}$ is the 8×8 k·p Hamiltonian, $H_{S}$ is the corresponding strain Hamiltonian and V(z) describes the conduction and valence band offsets. In this case, for active region QW of an ICL structure, a net strain was obtained from the relations

$$\varepsilon =\frac{\sum_{i}L_{i}\varepsilon_{i}}{\sum_{i}L_{i}}$$

where $L_{i}$ and $\varepsilon_{i}$ are the thicknesses of layers forming the given part of the active region and the non-zero components of the strain tensor in the layer, respectively. The carrier wave functions and the subband energies are determined by numerically solving the Schrödinger equation employing the finite difference method (FDM). The FDM is suitable for this kind of calculation because it is fast and facilitates using an arbitrary mesh [39]. To calculate the electronic structure and wave functions we used the standard mathematical subroutines available in the LAPACK libraries [40]. To investigate the optical transitions, we mainly examined the matrix elements, which are primarily determined by the spatial overlap integral of the electron and hole wave functions. More details on the calculations’ methodology can be found in Refs. [38,41]. All the material parameters were taken from Refs. [42,43] for 300 K. The total gain was obtained by using the subband-to-subband optical transitions and which is described in Refs. [41,44]. We assume that the half linewidth of the Lorentzian function is equal to 5 meV, based on the transport data for n- and p-type InAs/Ga_1−x_In_x_Sb superlattices of type II [45]. We present the results for TE polarization only.

## 3. Results

**Figure 1:**
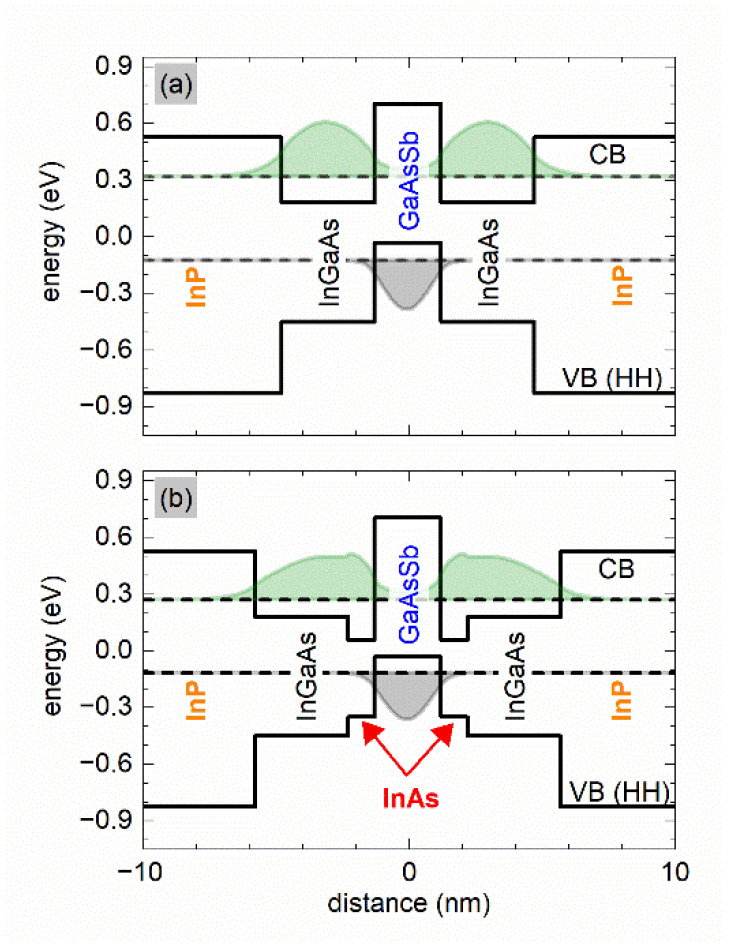
Energy band diagram and the squared waves function moduli for an (**a**) InP/In_0.7_Ga_0.3_As(3.5 nm)/GaAs_0.45_Sb_0.55_(2.5 nm)/In_0.7_Ga_0.3_As(3.5 nm)/InP (**b**) InP/In_0.7_Ga_0.3_As(3.5 nm)/InAs/GaAs_0.45_Sb_0.55_(2.5 nm)/InAs/In_0.7_Ga_0.3_As(3.5 nm)/InP type II “W” QW along the growth direction.

Figure 1a,b show the conduction and heavy-hole band edge profiles after including the strain, the fundamental electron e_1_ and heavy hole hh_1_ energy levels and the squared moduli of the corresponding wave functions (expressing the distribution of the probability densities) for two cases of the so called W-design structures (i.e., when using two QWs in the conduction band, such offer enhanced optical transition oscillator strengths while still keeping the type II lineup [5]). The first one is a strained type II QW, for which we chose the following compositions and thicknesses: In_0.7_Ga_0.3_As(3.5 nm)/GaAs_0.45_Sb_0.55_(2.5 nm)/In_0.7_Ga_0.3_As(3.5 nm) on InP substrate, Figure 1a (InP barriers). The calculated value of ε∗ is −0.96% in this case. The corresponding emission wavelength is about 2.82 µm at 300 K. As expected, the maxima of the squared wave function moduli can be found in the InGaAs and GaAsSb layers for electrons and holes, respectively.

In the second QW structure (Figure 1b), we propose to insert a thin InAs layer between the InGaAs and GaAsSb layers. Due to significant lattice mismatch between InAs and the InP substrate, and hence high strain, the used InAs layer thickness must be kept small, as it induces additional strain (here it is 1.0 nm). Comparing the structures presented in Figure 1a,b, we find that the calculated net strain changes from −0.96% to −1.35%, respectively. However, the advantage of the added InAs insertion is a significant red shift of the emission wavelength from 2.8 μm to 3.1 μm, which is targeted. This effect comes from changing the confinement for electrons via modifying the structure shown in Figure 1b. In particular, it can be observed that the addition of the InAs layer shifts the electron ground state down in energy and moves the maximum of the squared waves function moduli into the InAs layer. This in turn decreases the fundamental, type II optical transition energy and increases its intensity—both are beneficial in our case.

**Figure 2:**
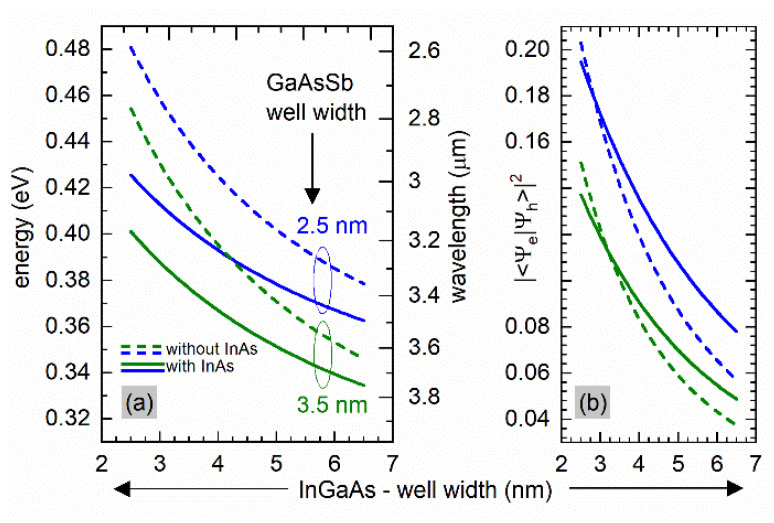
(**a**) Calculated ground state transition emission energy and the related overlap integrals (**b**) for InP/In_0.7_Ga_0.3_As/GaAs_0.45_Sb_0.55/_In_0.7_Ga_0.3_As/InP type II “W” (short-dash lines) and for and InP/In_0.7_Ga_0.3_As/InAs/GaAs_0.45_Sb_0.55_/InAs/In_0.7_Ga_0.3_As/InP type II “W” (solid lines). The thickness of the InAs layer was 1.0 nm.

Figure 2a,b present the calculated dependence of the active transition energy and the squared overlap integral of the electron and hole wave functions on InGaAs layer thickness in this kind of W-design QWs, for the cases as presented in from Figure 1, i.e., without and with the InAs layer, respectively, for two different thicknesses of the quaternary GaAsSb layer. First, we find that the entire MIR range demanded by the applications can be covered spectrally by emission from such QWs. Second, it is seen that spatially indirect type-II recombination allows for even longer emission wavelengths when the additional InAs layer is included (Figure 2a), with even enlarged transition oscillator strength in the longer wavelengths range (Figure 2b). However, as mentioned above, the additional InAs layer causes higher average strain. But to minimize this effect, GaAsSb material with increased antimony content can be taken into account. 

**Figure 3:**
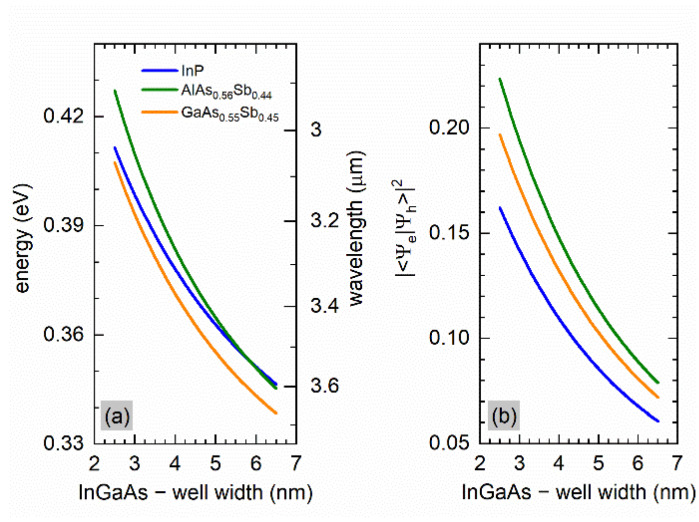
The fundamental transition energy (**a**) and the squared overlap integral (**b**) as a function of InGaAs well width in W-design (X)/In_0.7_Ga_0.3_As/InAs/GaAs_0.45_Sb_0.55/_/InAs/In_0.7_Ga_0.3_As/(X), where (X) = InP (blue solid line), AlAs_0.56_Sb_0.44_ (green solid line), GaAs_0.55_Sb_0.45_ (orange solid line). The thicknesses of the InAs and GaAs_0.45_Sb_0.55_ layers were 1.0 nm and 3.0 nm.

In Figure 3—similar to Figure 2—the calculated transition energy (wavelength) and overlap integrals are presented, but now it is for various external barrier materials (those directly surrounding the active regions in ICL structure). We consider just InP, but also two other alloys that are lattice-matched to InP, i.e., AlAs_0.56_Sb_0.44_ and GaAs_0.55_Sb_0.45_. It can be seen that with increasing width of the InGaAs quantum well, changing the barrier material from InP to AlAs_0.56_Sb_0.44_ does not bring the expected increase in emission wavelength. However, promising results are obtained when the surrounding of the active region is made of GaAs_0.55_Sb_0.45_. The effect of the type II structure modifications on the fundamental optical transitions oscillator strength, expressed by the squared overlap integral, is shown in Figure 3b. Particularly important is the fact that the transition intensity increases significantly for the latter barrier case. For comparison, in the case of structures grown on GaSb substrates, the transition intensity values (in sense of squared overlaps) are about 0.2 in a structure of AlSb/InAs(3.0 nm)/GaInSb(3.5 nm)/InAs(3.0 nm)/AlSb [38], about 0.3 for AlSb/InAs(3.0 nm)/GaAsSb(3.0 nm)/InAs(3.0 nm)/AlSb and about 0.2 for AlSb/InAs(3.0 nm)/GaAsSb(7.0 nm)/InAs(3.0 nm)/AlSb [46], and approx. 0.25 for the type-II W-design lattice-matched to GaSb [47]. This also holds true for the cases corresponding to longer wavelength emission, which shows directly the main advantage of using the materials proposed here to be deposited on the InP substrate.

**Figure 4:**
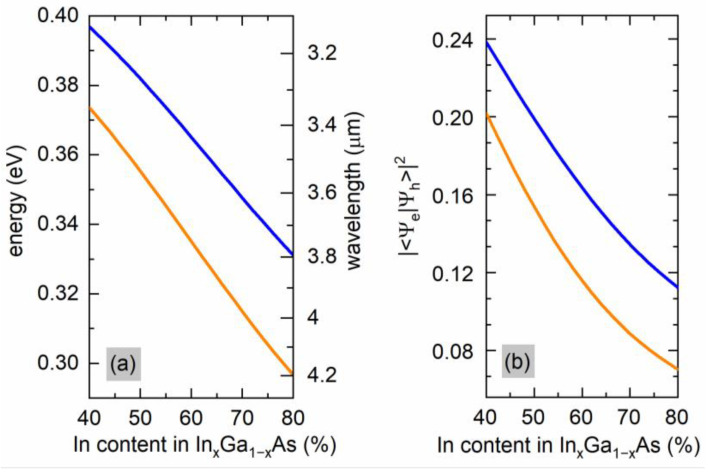
Emission energy (**a**) and the squared overlap integral (**b**) for an GaAs_0.55_Sb_0.45_/In_x_Ga_1−x_As/InAs(1.0 nm)/GaAs_0.45_Sb_0.55_(3.5 nm)/InAs(1.0 nm)/In_x_Ga_1−x_As/GaAs_0.55_Sb_0.45_ type II “W” (solid line) of varying In mole fraction in In_x_Ga_1−x_As. The blue (orange) lines correspond to a 3.0(4.5)-nm-thick In_x_Ga_1−x_As layer.

Figure 4 presents the calculated In content dependence of the e_1_–hh_1_ transition energy in the type-II W-design GaAs_0.55_Sb_0.45_/In_x_Ga_1−x_As/InAs(1.0 nm)/GaAs_0.45_Sb_0.55_(3.5 nm)/InAs(1.0 nm)/In_x_Ga_1−x_As/GaAs_0.55_Sb_0.45_ QW for two different In_x_Ga_1−x_As widths. First, Figure 4a shows that the change of the composition in the confinement layer affects the transition energy significantly because of shifting the electron energy levels (the hole ones are almost unaffected), and hence allows for easy tunability in the target range of the MIR. This is due to a combined effect of both; the band gap change of the material but also the strain—changing the In content in In_x_Ga_1−x_As in the considered range means increasing the in-plane strain from 0.6% to 1.4%. 

The effect of the type II structure modifications on the fundamental optical transitions oscillator strength is shown in Figure 4b. There can be mainly observed that the transition intensity decreases slightly when the In mole fraction increases from 40% to 80% in In_x_Ga_1−x_As (by a factor of 2–3). It can also be seen that when the In_x_Ga_1−x_As well width increases from 3.0 nm to 4.5 nm, the fundamental optical transitions oscillator strength increases, which can compensate for the intensity loss due to changes of the indium content. This shows the overall compositions and thicknesses range for possible tunability of this type of active region used in an ICL.

**Figure 5:**
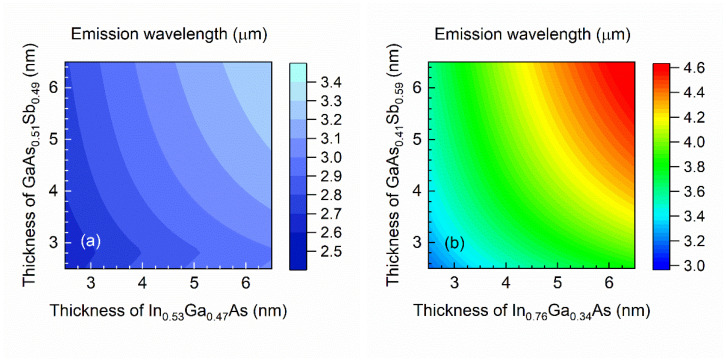
Contour plots of emission wavelength at the zone center for (**a**) GaAs_0.58_Sb_0.42_/In_0.53_Ga_0.47_As/InAs(1.0 nm)/GaAs_0.51_Sb_0.49_/InAs(1.0 nm)/In_0.53_Ga_0.47_As/GaAs_0.58_Sb_0.42_ and (**b**) GaAs_0.58_Sb_0.42_/In_0.76_Ga_0.24_As/InAs(1.0 nm)/GaAs_0.41_Sb_0.59_/InAs(1.0 nm)/In_0.76_Ga_0.24_As/GaAs_0.58_Sb_0.42_ type II “W” type-II ‘W’ QW structures at 300 K, versus electron and hole well thicknesses.

Then, we study the GaAs_0.58_Sb_0.42_/X/InAs(1.0 nm)/Y/InAs(1.0 nm)/X/GaAs_0.58_Sb_0.4_ system in a broader range of parameters in the type II W-structure on InP substrate (for X = In_0.53_Ga_0.47_As or In_0.76_Ga_0.24_As and Y = GaAs_0.49_Sb_0.51_ or GaAs_0.41_Sb_0.59_). Figure 5 illustrates a contour plot of the calculated emission wavelength dependence of the QW width in the conduction band (i.e., X-type material) versus valence band QW width (i.e., Y-type material). In the case when the material confining electrons is lattice matched to the substrate the emission wavelength reaches 3.5 µm, at most. A significantly different situation takes place when the confinement layer for electrons is made of a material that is compressively strained (net strain in the range from −0.98% to −1.23%)—then the emission wavelength can exceed 4 µm. 

Figure 5b also shows that the emission wavelength is further red shifted when an additional InAs layer is inserted into the QW structure. In order to maximize the shift, we slightly modified the materials within the active region—for a QW made of GaAs_0.58_Sb_0.42_/In_0.76_Ga_0.24_As/InAs/GaAs_0.41_Sb_0.59_/InAs/In_0.76_Ga_0.24_As/GaAs_0.58_Sb_0.42_ the obtained emission wavelength is in the range from about 3.0 μm to 4.6 µm.

**Figure 6:**
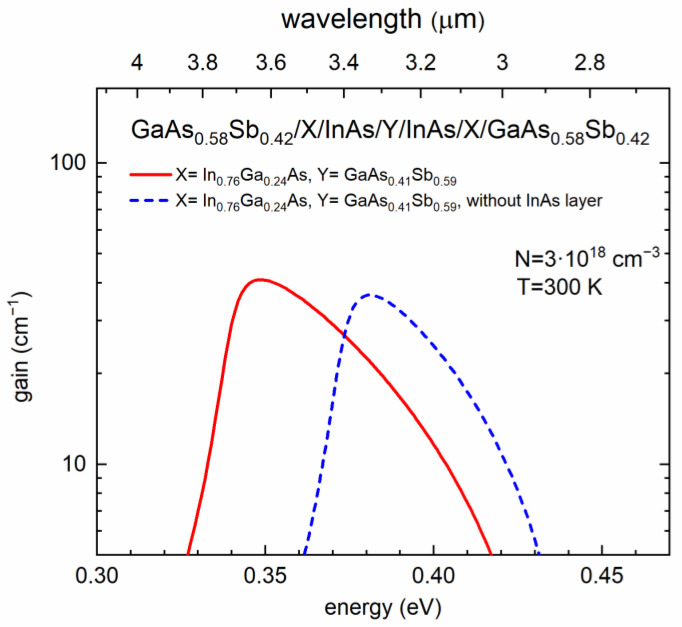
Calculated optical gain spectra for GaAs_0.58_Sb_0.42_/In_0.76_Ga_0.24_As(4.0 nm)/GaAs_0.41_Sb_0.59_(3.0 nm)/In_0.76_Ga_0.24_As(4.0 nm)/GaAs_0.58_Sb_0.42_ and GaAs_0.58_Sb_0.42_/In_0.76_Ga_0.24_As(4.0 nm)/InAs(1.0 nm)/GaAs_0.41_Sb_0.59_(3.0 nm)/InAs(1.0 nm)/In_0.76_Ga_0.24_As(4.0 nm)/GaAs_0.58_Sb_0.42_ “W” design type-II ‘W’ QW structures.

Eventually, for two selected cases, we have calculated the corresponding optical gain spectra (see Figure 6), i.e., for W-shaped QWs of GaAs_0.58_Sb_0.42_/In_0.76_Ga_0.24_As/GaAs_0.41_Sb_0.59_/In_0.76_Ga_0.24_As/GaAs_0.58_Sb_0.42_ without the InAs insertion (blue dashed line) and with the additional InAs layer (red solid line)—GaAs_0.58_Sb_0.42_/In_0.76_Ga_0.24_As/**InAs**/GaAs_0.41_Sb_0.59_/**InAs**/In_0.76_Ga_0.24_As/GaAs_0.58_Sb_0.42_ QW—the chosen layer thicknesses are given in the caption of Figure 6. The optical gain spectra have been calculated for an exemplary, but realistic injected carriers’ density of 3 × 10^18^ cm^−3^ at 300 K. The obtained maximum values of the gain equal about 40 cm^—1^, which is similar to those typically obtained for the type II active regions on GaSb or InAs substrates [28,45]. It is also clearly seen, in agreement with the discussion above, that adding the thin InAs layer increases the gain slightly and shifts it to longer wavelengths (by almost 0.4 μm in this particular case).

## 4. Conclusions

We have studied the influence of material configuration (compositions and layers’ thicknesses) on the electronic structure and the material gain of a type-II W-design QWs as the active region of an interband cascade laser, and considered their possible epitaxial deposition on InP substrates. The proposed original solution is based on a combination of InGaAs/GaAsSb materials, forming a broken gap layout, with an included additional InAs layer in the active part. We have chosen a set of parameters, including changes of the QW barrier material, which allow to combine the application-relevant spectral range with reasonably large transition oscillator strengths, and low enough net strain to assure the coherent growth without the strain relaxation. We have concentrated on the emission wavelengths of about 3–5 μm as it is the target range for many optical laser-based gas sensing applications and it is also one of the windows of the free space optical communication. These cause demands on new laser solutions with improved characteristics and lower fabrication costs. In that context, we have demonstrated for the first time that using the approach with particularly modified type II InGaAs/**InAs**/GaAsSb active region to be grown on an InP substrate should allow fabricating the interband cascade lasers with performances, which can be expected to be comparable to the state of the art devices on GaSb substrate, while also significantly cheaper and obtained in mature semiconductor technology. These translate into high structural and optical quality of the grown materials if only the strain is kept on an acceptable level, which we show is also possible. All these can be employed to fabricate MIR emitting InP-based ICLs, which have not been demonstrated so far. Such devices can directly be realized in practice in MBE or even MOCVD, as the growth of the materials under consideration is rather well-mastered, and hence it should be possible to also combine with the necessary injector layers and claddings. Therefore, we hope that our work will stimulate the experimental efforts, especially in the material development and device fabrication to grow such structures in order to verify our predictions, and will pave the way towards first real ICL devices compatible with InP substrates.

## Figures and Tables

**Table 1:** Summary of the current status on various approaches with QWs on InP substrate to reach the MIR emission.

Active Region	Emission Wavelength (μm)	Comments	References
“M”-type GaAsSb/InGaAs quantum-well	2.4–2.5	Room temperature PL at 2.5 μm and optically pumped lasing at 2.41 μm	[29]
W-shaped type II InGaAs/GaAsSb QWs	∼2.5	VCSEL device	[30]
Type I InAs/InGaAs/InAlAs QWs	∼2.9	Low-temperature lasing in pulsed mode; growth on In_0.8_Al_0.2_As metamorphic buffer	[31]
GaInAs/GaAsSb type-II quantum wells	2.6–3.9	Electrically pumped lasing in pulsed mode at 2.6 μm; spontaneous emission up to 3.9 μm	[32]
InGaAs/GaAsSb type-II ‘W-design’ quantum wells	> 2.0	Room temperature PL at ∼2.1 µm; up to ∼2.5 µm from calculations	[33]
GaInAs/GaAsSb type-II quantum wells	2.4–3.0	Room temperature PL data	[34]
Type II W-shaped InAsN/GaAsSb/InAsN/GaInP QW	∼3.8	Only theoretical modelling data	[28]
Type I QWs: GaInNAs/InP; GaNAsSb/InP; GaNPSb/InP	up to 3.6	Theoretical modelling of the gain	[35]
InGaAs/GaAsSbBi type II quantum wells	up to 3.26	Theoretical modelling of the gain	[36]
InGaAs/GaAsSbBi type I quantum wells	>3.0	Theoretical modelling	[37]

## Data Availability

The data that support the findings of this research are available from the corresponding author upon reasonable request.
